# Subtype-specific gout susceptibility loci and enrichment of selection pressure on *ABCG2* and *ALDH2* identified by subtype genome-wide meta-analyses of clinically defined gout patients

**DOI:** 10.1136/annrheumdis-2019-216644

**Published:** 2020-04-01

**Authors:** Akiyoshi Nakayama, Masahiro Nakatochi, Yusuke Kawamura, Ken Yamamoto, Hirofumi Nakaoka, Seiko Shimizu, Toshihide Higashino, Teruhide Koyama, Asahi Hishida, Kiyonori Kuriki, Miki Watanabe, Toru Shimizu, Keiko Ooyama, Hiroshi Ooyama, Mitsuo Nagase, Yuji Hidaka, Daisuke Matsui, Takashi Tamura, Takeshi Nishiyama, Chisato Shimanoe, Sakurako Katsuura-Kamano, Naoyuki Takashima, Yuya Shirai, Makoto Kawaguchi, Mikiya Takao, Ryo Sugiyama, Yuzo Takada, Takahiro Nakamura, Hiroshi Nakashima, Masashi Tsunoda, Inaho Danjoh, Atsushi Hozawa, Kazuyoshi Hosomichi, Yu Toyoda, Yu Kubota, Tappei Takada, Hiroshi Suzuki, Blanka Stiburkova, Tanya J. Major, Tony R. Merriman, Nagato Kuriyama, Haruo Mikami, Toshiro Takezaki, Keitaro Matsuo, Sadao Suzuki, Tatsuo Hosoya, Yoichiro Kamatani, Michiaki Kubo, Kimiyoshi Ichida, Kenji Wakai, Ituro Inoue, Yukinori Okada, Nariyoshi Shinomiya, Hirotaka Matsuo, On behalf of Japan Gout Genomics Consortium (Japan Gout), Katsuhisa Inoue

**Affiliations:** 1 Department of Integrative Physiology and Bio-Nano Medicine, National Defense Medical College, Tokorozawa, Japan; 2 Medical Squadron, Air Base Group, Western Aircraft Control and Warning Wing, Japan Air Self-Defense Force, Kasuga, Japan; 3 Division of Department of Nursing, Nagoya University Graduate School of Medicine, Nagoya, Japan; 4 Department of General Medicine, National Defense Medical College, Tokorozawa, Japan; 5 Department of Medical Biochemistry, Kurume University School of Medicine, Kurume, Japan; 6 Division of Human Genetics, Department of Integrated Genetics, National Institute of Genetics, Mishima, Japan; 7 Graduate School of Information Science and Technology, Hokkaido University, Sapporo, Japan; 8 Department of Epidemiology for Community Health and Medicine, Kyoto Prefectural University of Medicine, Kyoto, Japan; 9 Department of Preventive Medicine, Nagoya University Graduate School of Medicine, Nagoya, Japan; 10 Laboratory of Public Health, School of Food and Nutritional Sciences, University of Shizuoka, Shizuoka, Japan; 11 Department of Public Health, Nagoya City University Graduate School Medical Science, Nagoya, Japan; 12 Midorigaoka Hospital, Takatsuki, Japan; 13 Kyoto Industrial Health Association, Kyoto, Japan; 14 Ryougoku East Gate Clinic, Tokyo, Japan; 15 Nagase Clinic, Tokyo, Japan; 16 Akasaka Central Clinic, Tokyo, Japan; 17 Department of Preventive Medicine, Faculty of Medicine, Saga University, Saga, Japan; 18 Clinical Research Center, Saga University Hospital, Saga, Japan; 19 Department of Preventive Medicine, Institute of Biomedical Sciences, Tokushima University Graduate School, Tokushima, Japan; 20 Department of Health Science, Shiga University of Medical Science, Otsu, Japan; 21 Department of Public Health, Faculty of Medicine, Kindai University, Osaka-Sayama, Japan; 22 Department of Statistical Genetics, Osaka University Graduate School of Medicine, Suita, Japan; 23 Department of Respiratory Medicine and Clinical Immunology, Osaka University Graduate School of Medicine, Suita, Japan; 24 Department of Urology, National Defense Medical College, Tokorozawa, Japan; 25 Department of Surgery, National Defense Medical College, Tokorozawa, Japan; 26 Faculty of Medical Science, Teikyo University of Science, Tokyo, Japan; 27 Laboratory for Mathematics, National Defense Medical College, Tokorozawa, Japan; 28 Department of Preventive Medicine and Public Health, National Defense Medical College, Tokorozawa, Japan; 29 Group of Privacy Controls, Tohoku Medical Megabank Organization, Sendai, Japan; 30 Department of Preventive Medicine and Epidemiology, Tohoku Medical Megabank Organization, Tohoku University, Sendai, Japan; 31 Department of Bioinformatics and Genomics, Graduate School of Advanced Preventive Medical Sciences, Kanazawa University, Kanazawa, Japan; 32 Department of Pharmacy, The University of Tokyo Hospital, Tokyo, Japan; 33 Department of Pediatrics and Adolescent Medicine, First Faculty of Medicine, Charles University and General University Hospital, Prague, Czech Republic; 34 Institute of Rheumatology, Prague, Czech Republic; 35 Department of Biochemisty, University of Otago, Dunedin, New Zealand; 36 Cancer Prevention Center, Chiba Cancer Center Research Institute, Chiba, Japan; 37 Department of International Island and Community Medicine, Kagoshima University Graduate School of Medical and Dental Sciences, Kagoshima, Japan; 38 Division of Cancer Epidemiology and Prevention, Aichi Cancer Center Research Institute, Nagoya, Japan; 39 Department of Epidemiology, Nagoya University Graduate School of Medicine, Nagoya, Japan; 40 Division of Kidney and Hypertension, Department of Internal Medicine, Jikei University School of Medicine, Tokyo, Japan; 41 Department of Pathophysiology and Therapy in Chronic Kidney Disease, Jikei University School of Medicine, Tokyo, Japan; 42 Laboratory for Statistical Analysis, RIKEN Center for Integrative Medical Sciences, Yokohama, Japan; 43 Center for Genomic Medicine, Kyoto University Graduate School of Medicine, Kyoto, Japan; 44 RIKEN Center for Integrative Medical Sciences, Yokohama, Japan; 45 Department of Pathophysiology, Tokyo University of Pharmacy and Life Science, Hachioji, Japan; 46 Laboratory of Statistical Immunology, Immunology Frontier Research Center (WPI-IFReC), Osaka University, Suita, Japan

**Keywords:** gout/hyperuricaemia, genome-wide association study (GWAS), Japanese, subtype specific locus, selection pressure analysis

## Abstract

**Objectives:**

Genome-wide meta-analyses of clinically defined gout were performed to identify subtype-specific susceptibility loci. Evaluation using selection pressure analysis with these loci was also conducted to investigate genetic risks characteristic of the Japanese population over the last 2000–3000 years.

**Methods:**

Two genome-wide association studies (GWASs) of 3053 clinically defined gout cases and 4554 controls from Japanese males were performed using the Japonica Array and Illumina Array platforms. About 7.2 million single-nucleotide polymorphisms were meta-analysed after imputation. Patients were then divided into four clinical subtypes (the renal underexcretion type, renal overload type, combined type and normal type), and meta-analyses were conducted in the same manner. Selection pressure analyses using singleton density score were also performed on each subtype.

**Results:**

In addition to the eight loci we reported previously, two novel loci, *PIBF1* and *ACSM2B*, were identified at a genome-wide significance level (p<5.0×10^–8^) from a GWAS meta-analysis of all gout patients, and other two novel intergenic loci, *CD2-PTGFRN* and *SLC28A3-NTRK2*, from normal type gout patients. Subtype-dependent patterns of Manhattan plots were observed with subtype GWASs of gout patients, indicating that these subtype-specific loci suggest differences in pathophysiology along patients’ gout subtypes. Selection pressure analysis revealed significant enrichment of selection pressure on *ABCG2* in addition to *ALDH2* loci for all subtypes except for normal type gout.

**Conclusions:**

Our findings on subtype GWAS meta-analyses and selection pressure analysis of gout will assist elucidation of the subtype-dependent molecular targets and evolutionary involvement among genotype, phenotype and subtype-specific tailor-made medicine/prevention of gout and hyperuricaemia.

**Key messages**

**What is already known about this subject?**

- Our previous genome-wide association study (GWAS) was performed on broad subtypes of gout with only 945 gout cases. A recent study has revealed genetic adaptive evolution of gout in the Japanese population.

**What does this study add?**

- This is the first GWAS meta-analyses of clinically defined gout with more finely differentiated subtypes using two GWAS platforms with larger samples (3055 cases and 4554 controls). We identified multiple subtype-specific loci including four novel loci such as *CD2*, which encodes a well-known surface antigen found on all peripheral blood T-cells.
- The present study showed significant enrichment of selection pressure on two genes, *ABCG2* and *ALDH2*, for gout susceptibility in the Japanese population over the last 2000–3000 years.

**Key messages**

**How might this impact on clinical practice or future developments?**

- Our subtype GWASs of gout enabled us to develop subtype-dependent molecular targets that will lead to novel subtype-specific genome tailor-made therapies for gout/hyperuricaemia.
- The present study also elucidates the Japanese genetic evolution of susceptibility to gout/hyperuricaemia and its subtypes.

## Introduction

Gout is a well-known disease that manifests as acute and severe non-infectious arthritis.^1 2^ According to patients’ clinical parameters which reflect its causes,^2–5^ gout can be classified into four distinct subtypes: the renal underexcretion (RUE) type, renal overload (ROL) type, combined type and normal type, as shown in table 1 and online supplementary figure S1. Because these subtypes reflect causes of gout, genome-wide association studies (GWASs) of these subtypes are also likely to indicate its various genetic and pathophysiological backgrounds. While dividing patients into these subtypes is helpful for understanding patients’ pathophysiology, GWASs of these subtypes have only rarely been conducted, partly because clinical data, including time-consuming urinary collection, are necessary to categorise these subtypes. We previously performed a GWAS with clinically defined gout patients,^6^ followed by another with broader subtypes:^7^ RUE gout and ROL gout (table 1 and online supplementary figure S1), that revealed their specific loci. Although we were able to show these associations, this process has its limitations, including the use of a custom chip for replication studies that did not provide comprehensive genetic association searching. We use finely differentiated subtypes in daily clinical settings but there were not sufficient numbers of patients in the previous study^7^ to enable a GWAS with these finely differentiated subtypes. This prompted us to conduct, for the first time, GWASs with four distinct subtypes using meta-analysis across two GWAS platforms with a larger number of patients. We additionally conducted selection pressure analysis of the Japanese population on gout subtypes with the risk loci identified in the present study in order to investigate the evolutionary selective pressure on the Japanese population over the last 2000–3000 years.

10.1136/annrheumdis-2019-216644.supp1 Supplementary data

**Table 1:** Subtypes of gout* used in the present study

Subtype	Clinical parameters
Differentiated subtype	
RUE type gout	FE_UA_ <5.5% and UUE ≤25
ROL type gout	FE_UA_≥5.5% and UUE >25
Combined type gout	FE_UA_ <5.5% and UUE >25
Normal type gout	FE_UA_≥5.5% and UUE ≤25
Broader subtype	
RUE gout (RUE type gout +combined type gout)	FE_UA_ <5.5%
ROL gout (ROL type gout +combined type gout)	UUE >25

*Subtypes of hyperuricaemia can be classified in the same manner.

FE_UA_, fractional excretion of uric acid (unit: %); ROL, renal overload; RUE, renal underexcretion; UUE, urinary urate excretion (unit: mg/h/1.73 m^2^).

## Methods

### Study subjects and patients involvement

We performed subtype genome-wide meta-analyses based on two case–control data sets for gout that included the Japonica Array^8^ and Illumina Array platforms. Patients with known clinical parameters were recruited from Japanese male outpatients at gout clinics (see online supplementary methods). All 3104 cases were clinically diagnosed as having primary gout according to the criteria established by the American College of Rheumatology,^9^ and their subtypes were also diagnosed along with their clinical parameters as described previously^3 5 6^ (table 1 and online supplementary figure S1). As controls, 6081 individuals were assigned from Japanese male participants in the Japan Multi-Institutional Collaborative Cohort Study (J-MICC Study).^10 11^ This research was done without patient involvement (see online supplementary methods).

### Genotyping and imputation for the Japonica Array data set

A total of 1048 male clinically defined gout cases and 1179 male controls from the J-MICC Study^10 11^ were genotyped with the use of a Japonica SNP Array.^8^ The detail of quality control is described in online supplementary methods. This quality control filtering resulted in the selection of 1028 case subjects and 1167 control subjects as well as 603 009 single-nucleotide polymorphisms (SNPs). Prephasing and imputation were performed using SHAPEIT2^12^ and Minimac3,^13^ respectively. Postimputation quality control was also performed as described in the online supplementary methods. Ultimately, 1028 case subjects and 952 control subjects as well as 7 529 176 SNPs remained for the GWAS analysis.

### Genotyping and imputation for the Illumina Array data set

As case data, 2056 male gout cases subjects were genotyped with the use of HumanOmniExpress or HumanOmniExpressExome BeadChip Arrays (Illumina, San Diego, CA, USA). The detail of quality control is described in the online supplementary methods. This quality control filtering resulted in the selection of 2032 case subjects and 4901 control subjects as well as 553 321 SNPs. Postimputation quality control was also performed as described in the online supplementary methods. Ultimately, 2025 case subjects and 3602 control subjects as well as 7 356 207 SNPs remained for the GWAS analysis.

### Association analysis for SNPs and gout

The association of SNPs with gout was assessed using logistic regression analysis (generalised linear model); the dependent variable was gout label (case=1, control=0), and the independent variables included imputed genotypes of each SNP and covariates. The covariates comprised the first four principal component scores. The effect sizes and standard errors estimated in logistic regression analysis were used in the subsequent meta-analysis. The association analysis was performed with the use of Efficient and Parallelizable Association Container Toolbox (EPACTS). https://genome.sph.umich.edu/wiki/EPACTS).

### Meta-analyses

The meta-analyses were performed using a total of 3053 cases and 4554 controls from the two data sets (online supplementary table S1–S3). The association results for each SNP across the studies were combined with METAL software^14^ using the fixed-effects inverse-variance-weighted method. Heterogeneity of effect sizes was assessed via the *I* ^2^ index. The meta-analysis included 7 206 774 SNPs and the results from both the Japonica and Illumina Arrays. The genome-wide significance level α was set to a p value of <5×10^–8^.

### Genetic correlation analysis

Genetic correlation analysis using linkage disequilibrium score (LDSC) regression analysis^15^ was conducted to examine the potential genetic overlap between gout subtypes and between each subtype and serum uric acid (SUA) levels. For the regression, we used the 1000 genomes phase_3 East Asian LDSC and summary statistics for high-quality common SNPs present in the HapMap 3 reference panel for each analysis.

### Selection pressure analysis

The details of genome-wide recent natural selection signature using singleton density score (SDS)^16^ from high-depth whole genome sequence data of the Japanese population had been described in the previous study.^17^ Using the same approach,^17^ we calculated the SDS of gout-risk variants identified in the present study, and evaluated overlaps between enrichment of the natural selection signatures and these variants from each subtype.

## Results

### Subtype GWASs of gout

**Figure 1:**
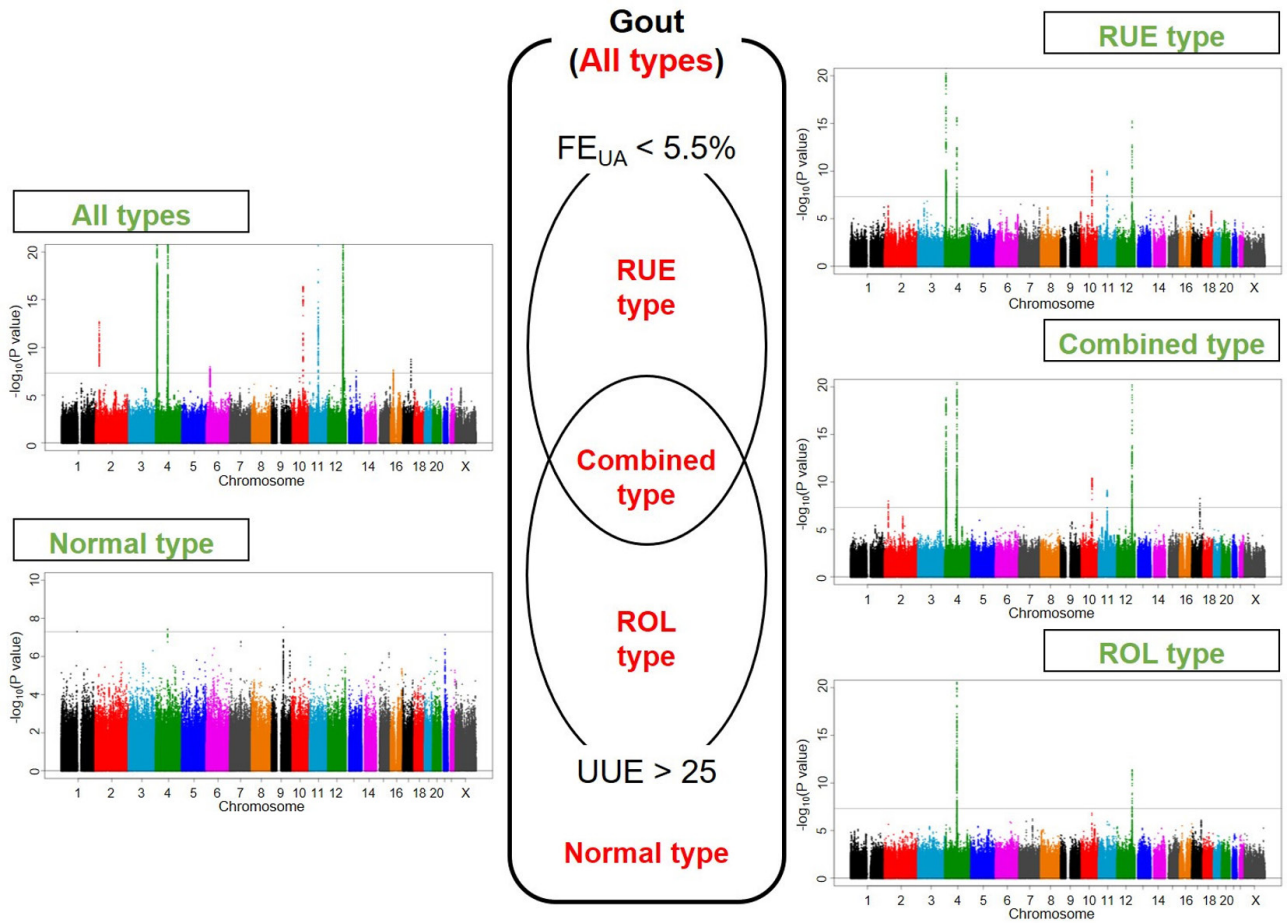
Manhattan plots of GWASs of subtypes of gout. Clinical subtypes and Manhattan plots of GWASs of all gout types, RUE type gout, combined type gout, ROL type gout and normal type gout are shown. The x-axis represents chromosomal positions and the y-axis shows −log_10_ p values. The dotted lines indicate the genome-wide significance threshold (p=5.0×10^–8^). FE_UA_, fractional excretion of uric acid (%); GWASs, genome-wide association studies; ROL, renal overload; RUE, renal underexcretion; UUE, urinary urate excretion (mg/h/1.73 m^2^). See table 1 and online supplementary figure S1 for a detailed classification of gout/hyperuricaemia.

**Figure 2:**
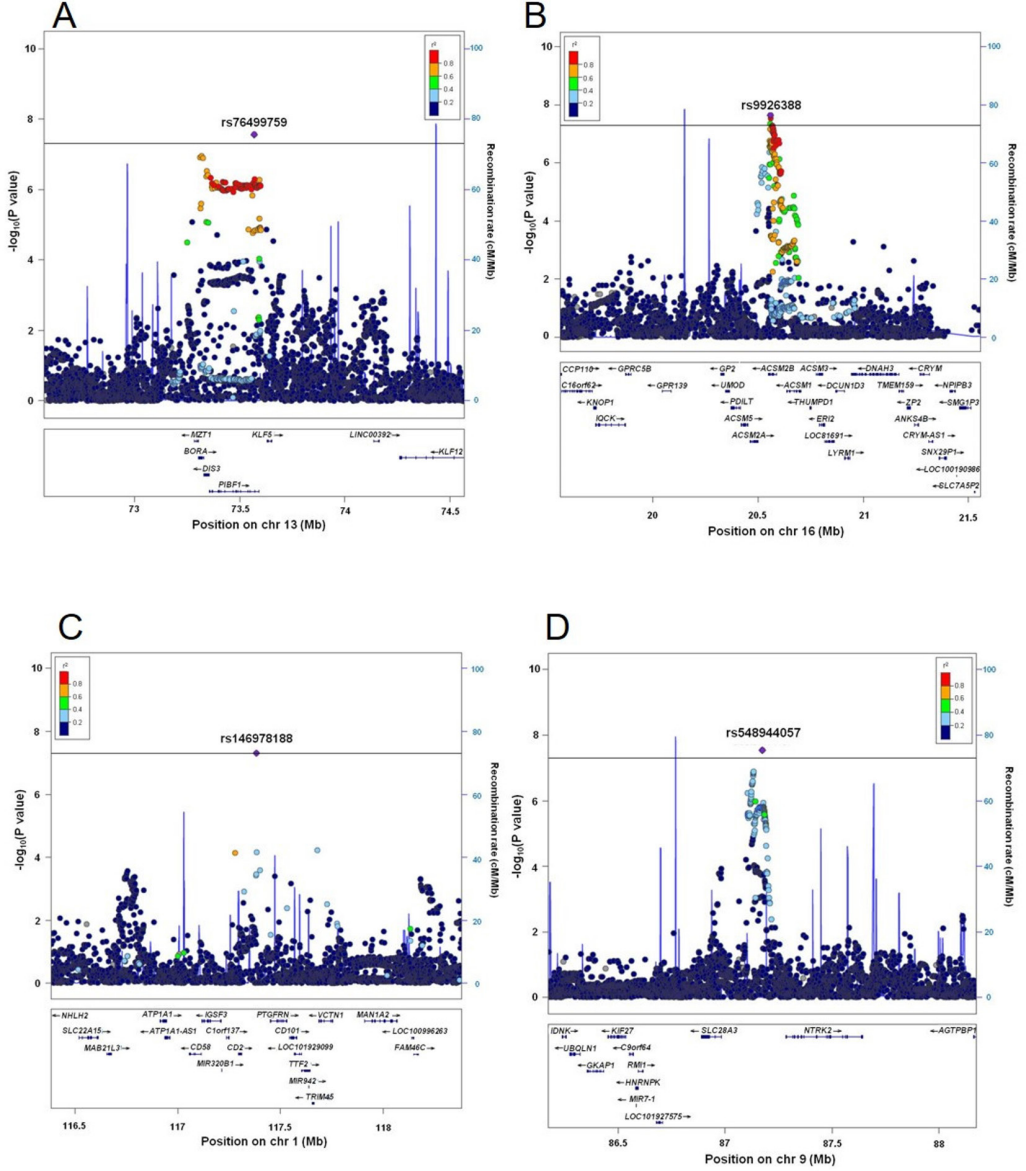
Regional association plots of novel gout loci. Two loci were revealed to exceed the genome-wide significance level from the meta-analysis of GWASs from all gout patients, and another two loci from normal type gout patients. The highest association signal in each panel is located on (A) *PIBF1*, (B) *ACSM2B*, (C) *CD2-PTGFRN* and (D) *SLC28A3-NTRK2*. The region within 1 Mb from the single-nucleotide polymorphism (SNP) indicating the lowest p value is shown. (Upper panel) Plots of −log_10_ p values for the test of SNP association with gout. The SNP showing the lowest p value in the meta-analysis is depicted as a purple diamond. Other SNPs are colour-coded according to the extent of linkage disequilibrium (measured in r^2^) with the SNP showing the lowest p value. Recombination rates (centimorgans per Mb) estimated from HapMap Phase II data are also plotted. (Lower panel) RefSeq genes. Genomic coordinates are based on NCBI human genome reference sequence build hg19. The r^2^ data were calculated with 1000 Genomes Project Phase_3 JPT samples.^45^ GWASs, genome-wide association studies.

Figure 1 displays Manhattan plots of all and four clinical subtypes of gout, and figure 2 shows regional plots of novel loci. Compared with the plotted pattern for all clinically defined gout patients, each gout subtype (RUE type, ROL type, combined type and normal type) shows a subtype-specific plotted pattern and indicates the presence of cause-specific associated genes such as *SLC2A9* and *ABCG2*. Table 2 lists the genome-wide significant loci from subtype GWASs. In total, 10, five, two, seven and three loci were identified at the genome-wide significant level to be associated with all, the RUE type, ROL type, combined type and normal type gout, respectively. Of these, two loci from all gout types, rs76499759 of *PIBF1* and rs9926388 of *ACSM2B*, and another two intergenic loci from normal type gout, rs146978188 of *CD2-PTGFRN* and rs548944057 of *SLC28A3-NTRK2*, were detected as novel loci.

**Table 2:** Significant gout loci identified in the present genome-wide meta-analyses

SNP*	Locus	Chr.	Position (bp)†	Gene‡	Alleles		Illumina Array				Japonica Array				Meta-analysis			
					Risk	Non- risk	RAF		OR (95% CI)	P value	RAF		OR (95% CI)	P value	OR (95% CI)	P value	I^2^	HetP
							Case	Control			Case	Control						
All gout patients																		
rs1260326	2p23.3	2	27 730 940	*GCKR*	T	C	0.623	0.545	1.33 (1.22 to 1.44)	8.75×10^–12^	0.613	0.565	1.22 (1.07 to 1.39)	3.61×10^–3^	1.30 (1.21 to 1.39)	2.07×10^–13^	10.3	0.291
rs3775946	4p16.1	4	9 995 256	*SLC2A9*	G	A	0.677	0.569	1.62 (1.49 to 1.76)	4.55×10^–29^	0.672	0.555	1.67 (1.46 to 1.92)	9.42×10^–14^	1.63 (1.52 to 1.75)	3.73×10^–41^	0	0.669
rs4148155	4q22.1	4	89 054 667	*ABCG2*	G	A	0.454	0.277	2.18 (2.00 to 2.38)	1.05×10^–70^	0.462	0.264	2.38 (2.06 to 2.75)	8.13×10^–33^	2.23 (2.08 to 2.41)	1.81×10^–101^	3.8	0.308
rs2817188	6p22.2	6	25 807 603	*SLC17A1*	G	A	0.874	0.825	1.33 (1.18 to 1.51)	5.03×10^–6^	0.871	0.836	1.40 (1.16 to 1.69)	5.03×10^–4^	1.35 (1.22 to 1.50)	1.06×10^–8^	0	0.660
rs3129500	10q23.2	10	88 915 107	*SHLD2/FAM35A*	G	A	0.423	0.358	1.40 (1.29 to 1.53)	2.36×10^–14^	0.423	0.371	1.30 (1.13 to 1.50)	2.71×10^–4^	1.37 (1.28 to 1.48)	4.34×10^–17^	0	0.355
rs145954970	11q13.1	11	64 273 830	*SLC22A11*	C	G	0.995	0.971	10.43 (5.83 to 18.66)	2.78×10^–15^	0.997	0.967	25.09 (7.83 to 80.37)	5.75×10^–8^	12.43 (7.39 to 20.92)	2.25×10^–21^	42.8	0.186
rs671	12q24.12	12	112 241 766	*ALDH2*	G	A	0.823	0.725	1.89 (1.71 to 2.08)	8.65×10^–36^	0.821	0.684	2.04 (1.75 to 2.37)	2.78×10^–20^	1.93 (1.78 to 2.10)	3.19×10^–54^	0	0.412
**rs76499759**	**13q22.1**	**13**	73 568 511	***PIBF1***	**A**	**G**	**0.219**	**0.180**	**1.30 (1.17 to 1.43**)	**2.56×10^–7^**	**0.212**	**0.183**	**1.20 (1.02 to 1.41**)	**2.62×10^–2^**	**1.27 (1.17 to 1.38**)	**2.79×10^–8^**	**0**	0.418
**rs9926388**	**16p12.3**	**16**	20 558 441	***ACSM2B***	**A**	**G**	**0.301**	**0.252**	**1.24 (1.14 to 1.36**)	**1.05×10^–6^**	**0.315**	**0.277**	**1.22 (1.06 to 1.42**)	**6.46×10^–3^**	**1.24 (1.15 to 1.33**)	**2.30×10^–8^**	**0**	0.861
rs1010269	17q23.2	17	59 448 945	*BCAS3*	G	A	0.558	0.503	1.24 (1.14 to 1.34)	6.06×10^–7^	0.583	0.528	1.26 (1.10 to 1.43)	7.68×10^–4^	1.24 (1.16 to 1.33)	1.81×10^–9^	0	0.836
RUE type gout patients																		
rs3775948	4p16.1	4	9 995 182	*SLC2A9*	C	G	0.705	0.573	1.83 (1.57 to 2.14)	2.60×10^–14^	0.717	0.556	2.04 (1.61 to 2.59)	3.82×10^–9^	1.89 (1.66 to 2.15)	8.01×10^–22^	0	0.448
rs4148155	4q22.1	4	89 054 667	*ABCG2*	G	A	0.388	0.277	1.66 (1.43 to 1.93)	2.72×10^–11^	0.382	0.264	1.74 (1.39 to 2.19)	1.73×10^–6^	1.69 (1.49 to 1.91)	2.54×10^–16^	0	0.737
rs9420434	10q23.2	10	88 843 209	*GLUD1 (SHLD2*)	C	T	0.316	0.245	1.49 (1.27 to 1.74)	6.39×10^–7^	0.342	0.243	1.65 (1.31 to 2.09)	2.37×10^–5^	1.54 (1.35 to 1.75)	8.62×10^–11^	0	0.462
rs76741582	11q13.1	11	64 247 850	*SLC22A11*	T	C	0.028	0.009	3.57 (2.16 to 5.88)	6.31×10^–7^	0.036	0.008	4.87 (2.31 to 10.25)	3.09×10^–5^	3.93 (2.59 to 5.95)	1.06×10^–10^	0	0.497
rs4646776	12q24.12	12	112 230 019	*ALDH2*	G	C	0.819	0.722	1.88 (1.57 to 2.26)	1.73×10^–11^	0.796	0.680	1.82 (1.4 to 2.36)	6.66×10^–6^	1.86 (1.60 to 2.16)	5.80×10^–16^	0	0.839
ROL type gout patients																		
rs4148155	4q22.1	4	89 054 667	*ABCG2*	G	A	0.515	0.277	2.87 (2.43 to 3.39)	5.14×10^–35^	0.493	0.264	2.61 (2 to 3.41)	2.05×10^–12^	2.79 (2.42 to 3.22)	9.75×10^–46^	0	0.559
rs11066008	12q24.12	12	112 140 669	*ACAD10 (ALDH2*)	A	G	0.724	0.643	1.67 (1.38 to 2.04)	2.49×10^–7^	0.751	0.600	2.12 (1.56 to 2.89)	1.57×10^–6^	1.79 (1.52 to 2.11)	4.20×10^–12^	39	0.200
Combined type gout patients																		
rs1260326	2p23.3	2	27 730 940	*GCKR*	T	C	0.647	0.545	1.45 (1.27 to 1.66)	4.92×10^–8^	0.615	0.565	1.23 (1.03 to 1.48)	2.50×10^–2^	1.37 (1.23 to 1.53)	1.05×10^–8^	50.5	0.155
rs3775948	4p16.1	4	9 995 182	*SLC2A9*	C	G	0.690	0.573	1.70 (1.48 to 1.95)	9.54×10^–14^	0.672	0.556	1.63 (1.35 to 1.97)	2.58×10^–7^	1.67 (1.50 to 1.87)	1.43×10^–19^	0	0.748
rs74904971	4q22.1	4	89 050 026	*ABCG2*	A	C	0.474	0.276	2.42 (2.11 to 2.78)	2.40×10^–36^	0.474	0.264	2.56 (2.11 to 3.09)	5.87×10^–22^	2.46 (2.20 to 2.76)	1.53×10^–56^	0	0.646
rs6586063	10q23.2	10	88 949 045	*SHLD2/FAM35A*	G	A	0.457	0.368	1.60 (1.39 to 1.85)	1.60×10^–10^	0.436	0.393	1.28 (1.04 to 1.58)	1.94×10^–2^	1.49 (1.32 to 1.68)	4.30×10^–11^	65.6	0.088
rs11231879	11q13.1	11	64 581 645	*CDC42BPG (SLC22A12*)	G	A	0.360	0.295	1.38 (1.19 to 1.60)	2.44×10^–5^	0.375	0.282	1.56 (1.29 to 1.89)	4.39×10^–6^	1.44 (1.28 to 1.62)	7.66×10^–10^	4.4	0.307
rs116873087	12q24.13	12	112 511 913	*NAA25 (ALDH2*)	G	C	0.828	0.749	2.21 (1.80 to 2.72)	5.86×10^–14^	0.845	0.683	2.61 (2.06 to 3.31)	2.69×10^–15^	2.38 (2.03 to 2.78)	1.88×10^–27^	6.1	0.302
rs9905274	17q23.2	17	59 450 441	*BCAS3*	C	T	0.558	0.482	1.34 (1.18 to 1.53)	1.20×10^–5^	0.581	0.499	1.42 (1.19 to 1.70)	1.03×10^–4^	1.37 (1.23 to 1.52)	5.62×10^–9^	0	0.596
Normal type gout patients																		
**rs146978188**	**1p13.1**	**1**	117 383 166	***CD2 - PTGFRN***	**A**	**G**	**0.065**	**0.015**	**6.53 (3.09 to 13.77**)	**8.35×10^–7^**	**0.086**	**0.021**	**5.58 (1.33 to 23.46**)	**1.90×10^–2^**	**6.31 (3.26 to 12.24**)	**4.93×10^–8^**	**0**	**0.849**
rs4148155	4q22.1	4	89 054 667	*ABCG2*	G	A	0.473	0.277	2.38 (1.71 to 3.33)	3.35×10^–7^	0.438	0.264	2.14 (1.05 to 4.36)	3.67×10^–2^	2.34 (1.73 to 3.16)	3.64×10^–8^	0	0.787
**rs548944057**	**9q21.33**	**9**	87 174 107	***SLC28A3 - NTRK2***	**T**	**A**	**0.125**	**0.046**	**3.89 (2.22 to 6.83**)	**2.13×10^–6^**	**0.160**	**0.047**	**4.58 (1.63 to 12.83**)	**3.83×10^–3^**	**4.04 (2.47 to 6.62**)	**2.91×10^–8^**	**0**	**0.788**

*dbSNP rs number.

†SNP positions are based on NCBI human genome reference sequence Build hg19.

‡Novel loci are shown in bold.

Chr, chromosome; RAF, risk allele frequency; ROL, renal overload; RUE, renal underexcretion; SNP, single-nucleotide polymorphism.

For all gout cases (table 2), 10 loci showed association at the genome-wide significance level: rs4148155 of *ABCG2* (p_meta_=1.81×10^–101^; ORs=2.23), rs671 of *ALDH2* (p_meta_=3.19×10^–54^; OR=1.93), rs3775946 of *SLC2A9* (p_meta_=3.73×10^–41^; OR=1.63), rs145954970 of *SLC22A11* (p_meta_=2.25×10^–21^; OR=12.43), rs3129500 of *FAM35A* (recently renamed as *SHLD2*, p_meta_=4.34×10^–17^; OR=1.37), rs1260326 of *GCKR* (p_meta_=2.07×10^–13^; OR=1.30), rs1010269 of *BCAS3* (p_meta_=1.81×10^–9^; OR=1.24), rs2817188 of *SLC17A1* (p_meta_=1.06×10^–8^; OR=1.35), rs9926388 of *ACSM2B* (p_meta_=2.30×10^–8^; OR=1.24) and rs76499759 of *PIBF1* (p_meta_=2.79×10^–8^; OR=1.27). Among these 10 loci, *PIBF1* and *ACSM2B* (table 2 and figure 2A, B). were identified for the first time as gout-risk loci at the genome-wide significance level. *BCAS3* was identified here for the first time by the GWAS approach with Japanese individuals, while Li *et al*^18^ reported that rs11653176, another SNP of *BCAS3*, is associated with gout based on a GWAS with a Han Chinese population. We also replicated its association with gout in a Japanese population using a candidate gene approach.^19^ Other loci have been previously reported to have an association with gout in our previous GWASs^6 7^ and association studies.^20 21^ As suggestive loci for gout, seven loci: *PDZK1*, *TACR1-EVA1A*, *LOC100128993*, *ARID5B*, *TOLLIP-AS1-BRSK2*, *SLC38A1* and *MLXIP*, were detected (see online supplementary table S4). Of these, *PDZK1*, a gene encoding a scaffolding protein^22 23^ such as for urate transporters SLC22A12/URAT1 and ABCG2, was reported to have an association with SUA by a GWAS approach^24 25^ and with gout as a result of candidate gene approach studies.^26–28^

As shown in online supplementary table S2, all the 3053 cases were classified into RUE type gout (654 cases), ROL type gout (486 cases), combined type gout (905 cases) and normal type gout (92 cases) for GWASs of gout subtypes.

The meta-analysis of a GWAS of the RUE type gout (table 2) showed significant SNPs in the following five loci: rs3775948 of *SLC2A9* (p_meta_=8.01×10^–22^; OR=1.89), rs4148155 of *ABCG2* (p_meta_=2.54×10^–16^; OR=1.69), rs4646776 of *ALDH2* (p_meta_=5.80×10^–16^; OR=1.86), rs9420434 of *GLUD1* (p_meta_=8.62×10^–11^; OR=1.54) and rs76741582 of *SLC22A11* (p_meta_=1.06×10^–10^; OR=3.93). Since *GLUD1* is in LD with *SHLD2/FAM35A*, for which we previously showed a significant association with gout,^7^ all of these five loci were previously identified as having an association with gout.^6 7^ We also detected nine suggestive loci: *SMYD3*, *GCKR-C2orf16*, *SMARCC1*, *FRMD4B-MITF*, *ARL4A-ETV1*, *C7orf66-EIF3IP1*, *ASB10*, *PXDNL* and *LOC100287896-POLD3*, for RUE type gout as shown in online supplementary table S4.

From ROL type gout (table 2), rs4148155 of *ABCG2* (p_meta_=9.75×10^–46^; OR=2.79) and rs11066008 of *ACAD10* (*ALDH2*) (p_meta_=4.20×10^–12^; OR=1.79) were revealed to have an association. We had previously reported both to have a significant association with gout.^6 7 20 21^ Three suggestive loci, *CNPY4*, *GRID1* and *KCNJ2-CASC17*, were also identified from ROL type gout (see online supplementary table S4).

Combined type gout displayed the following seven significant loci (table 2): rs74904971 of *ABCG2* (p_meta_=1.53×10^–56^; OR=2.46), rs116873087 of *NAA25* (p_meta_=1.88×10^–27^; OR=2.38), rs3775948 of *SLC2A9* (p_meta_=1.43×10^–19^; OR=1.67), rs6586063 of *SHLD2/FAM35A* (p_meta_=4.30×10^–11^; OR=1.49), rs9905274 of *BCAS3* (p_meta_=5.62×10^–9^; OR=1.37), rs11231879 of *CDC42BPG* (p_meta_=7.66×10^–10^; OR=1.44) and rs1260326 of *GCKR* (p_meta_=1.05×10^–8^; OR=1.37). There are studies on these which report an association between *CDC42BPG* and hyperuricaemia from the Japanese exome-wide association study,^29^ and GWAS on SUA from a Korean population.^30^ The significance around rs11231879 of *CDC42BPG* was, however, no longer evident when conditioned on rs11231879 itself, nor when conditioned on the secondarily significant SNP, rs56093838 of *SLC22A12/URAT1*, demonstrating that these signals were from the same locus (see online supplementary figure S2). Because URAT1 is a well-known urate transporter that markedly affects SUA level, these findings indicate that the true associated gene for combined type gout on chromosome 11q13.1 locus is not *CDC42BPG*, but *SLC22A12/URAT1*. rs116873087 of *NAA25* is in strong LD with rs671 of *ALDH2* (r^2^=0.97 in the 1000 Genomes Project Phase_3: JPT samples). All of the seven loci had therefore been previously identified as having an association with gout.^6 7 20 21^ Two suggestive loci, *NCKAP5-MIR3679* and *PRDM8-FGF5*, were also identified from combined type gout (see online supplementary table S4).

Three significant loci were found from normal type gout: rs548944057 of *SLC28A3-NTRK2* (p_meta_=2.91×10^–8^; OR=4.04), rs4148155 of *ABCG2* (p_meta_=3.64×10^–8^; OR=2.34) and rs146978188 of *CD2-PTGFRN* (p_meta_=4.93×10^–8^; OR=6.31). Of these, two intergenic loci are novel susceptibility loci for gout (table 2 and figure 2C, D) There were eight suggestive loci: *ZNF639-MFN1*, *RUNX2-CLIC5*, *DST*, *HGF*, *MED27-NTNG2*, *LINC00944-LINC02372*, *SV2B* and *GABPA*, for normal type gout as shown in online supplementary table S4.

The LDSC regression analysis was performed to examine the potential genetic overlap between gout subtypes and between each subtype and SUA levels. Significant positive genetic correlations were observed among these subtypes as well as between these traits and SUA levels (see online supplementary figure S3).

### Selection pressure analysis of gout susceptibility

**Figure 3:**
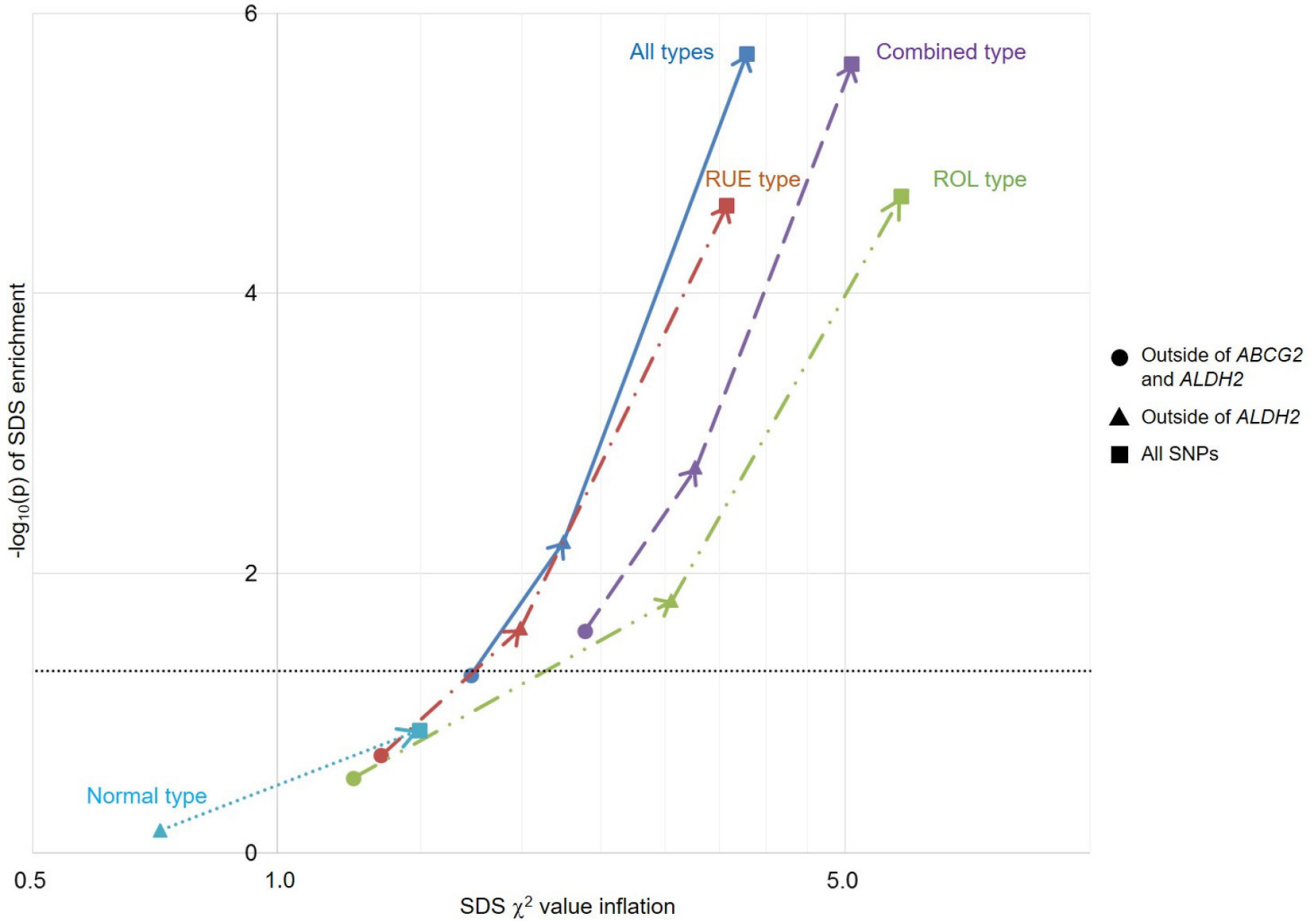
Overlap between natural selection signatures and genetic risk of gout and its subtypes in the Japanese population. For each trait, inflation of the selection χ^2^ value is indicated along the x-axis, and −log_10_(p) of enrichment is plotted along the y-axis. The horizontal grey line represents significance threshold (p<0.05). Because *ABCG2* and *ALDH2* are associated with a well-known genetic risk of gout in Japanese individuals, selection pressure analyses without these loci were performed initially (filled circle), and subsequent analyses were conducted with *ABCG2* (filled triangle), as well as *ABCG2* and *ALDH2* (filled square). When calculated with *ABCG2* (outside of *ALDH2*) (filled triangle), all but normal type gout showed significant selective pressure, indicating that *ABCG2* is involved in adaptive evolution in Japanese for having higher SUA levels, which can result in gout. Finally, all but normal type gout also showed significant selective pressure with *ABCG2* and *ALDH2* loci (filled square). ROL, renal overload; RUE, renal underexcretion; SDS, singleton density score; SNPs, single-nucleotide polymorphisms.

We also performed selection pressure analysis of gout on the basis of a previous report on the recent natural selection signature in the Japanese population.^17^ This analysis enables us to elucidate the genetic risks of gout characterised in the recent evolutionary history (2000–3000 years) of the Japanese population. Because *ALDH2* was reported to be subjected to strong selection pressure in the Japanese population,^17^ and because *ABCG2*,^31 32^ as well as *ALDH2*,^20 21 33^ is a well-known strong susceptible gene for gout in Japanese, selection pressure analysis was initially performed outside of these two loci. As a result, only combined type gout showed significant enrichment of selection pressure (p=0.026; figure 3 and online supplementary table S5). When the *ABCG2* locus was included in the analysis, all of these subtypes except for normal type gout then showed significant enrichment of selection pressure. As expected, analysis including *ALDH2* and outside of *ABCG2* showed significant enrichment of selection pressure except for normal type gout. This trend also persisted in the analysis with all associated SNPs (figure 3 and online supplementary table S5).

## Discussion

We previously performed GWASs of clinically defined gout cases in the Japanese population and found loci including *ABCG2*, *SLC2A9*, *ALDH2* (*CUX2*), *GCKR* and *SHLD2/FAM35A* to be associated with gout at a genome-wide significant level.^6 7^ While our previous study was performed with broader subtypes, it is one of the unique points of this study that the present GWAS is the first to be conducted with four differentiated subtypes: the RUE type, ROL type, combined type and normal type gout (figure 1), which are commonly used in daily clinical settings. Two platforms for GWASs and meta-analyses between them were used to perform a comprehensive genetic association search.

The results allowed four novel loci to be identified from the present study. The pathophysiological associations of the two novel loci from all gout types, *PIBF1* and *ACSM2B* with gout, are totally unknown. PIBF (progesterone-induced blocking factor) is induced by progesterone and is a mediator that exerts substantial antiabortive activities, including cytokine secretion.^34^ *PIBF1* might be therefore involved in decreased urate production by female hormone and/or decreased inflammatory response to gout attack. ACSM2B (acyl-CoA synthetase medium chain family member 2B) is a predominant transcript in the human liver and an enzyme catalysing the activation of medium-chain length fatty acids.^35^ Because ASCM2B is involved in the production of ATP, a purine body metabolised to urate, *ASCM2B* might contribute to gout via that mechanism. While the present study showed significance at SNPs of two genes, it is of course possible that these are mere markers and that the true risk SNPs are present close by. For example, since *UMOD*, a causative gene of uromodulin-associated kidney disease (previously known as familial juvenile hyperuricemic nephropathy)^36^ is located 180 kb downstream from rs9926388 of *ACSM2B*, there might be a relationship between them.

Another two intergenic loci from normal type gout, rs146978188 of *CD2-PTGFRN* and rs548944057 of *SLC28A3-NTRK2*, were detected for the first time to have an association with gout. CD2 is well known as a surface antigen found on all peripheral blood T-cells, and *PTGFRN/CD9P-1* encodes prostaglandin F2 receptor inhibitor. Neither of these was previously known to have an association with gout or uric acid. *CD101*, which is next to *PTGFRN* (150 kb downstream from rs146978188), is reported to be expressed on macrophages/monocytes and T-cells, to confer a modulatory/coregulatory function, and to be conspicuously downregulated in rheumatoid arthritis patients.^37^ Because macrophages are a chief contributor to gouty attack, CD101 might be the true susceptible gene for normal type gout. rs146978188 is also in LD with an SNP of *SLC22A15/FLIPT1*, an orphan transporter gene in the same family as *SLC22A12/URAT1*, a well-known urate transporter gene that is strongly associated with gout. This transporter gene might have an association with normal type gout. SLC28A3/CNT3 is reported to be an Na^+^-dependent pyrimidine-selective and purine-selective transporter found predominantly in the intestine and kidney,^38 39^ which are the main urate excretion pathways. Further analysis is needed to elucidate the relationship between this transporter gene and gout, including normal type gout.

While the present study revealed only suggestive loci from other subtype GWASs, some of these loci from subtype gout also suggest a relationship with RUE, extra-RUE and/or overproduction of urate, because gout subtypes reflect their clinical parameters (table 1). GWASs with these subtypes should therefore be useful for estimating the expression and function of proteins encoded by identified loci. rs557868370 of *SLC38A1*, a transporter gene, was detected as a suggestive locus (see online supplementary table S4). Since there is a study reporting the relationship between oxidative stress and SLC38A1/SNAT1,^40^ it might have a relationship with urate, which also has antioxidative stress effects. Because genetic variants in urate transporter genes such as *ABCG2*, *SLC2A9* and *SLC22A12* are well known to cause SUA variation and gout, it is also possible that the SLC38A1/SNAT1 transporter is involved in urate or purine transport.

Very recently, Tin *et al*^41^ performed transancestry GWAS meta-analysis of SUA and gout, including self-reported gout cases. We compared these SNPs and found five of the 10 loci detected here (*GCKR, SLC2A9, ABCG2, SLC17A1* and *BCAS3*) to be associated with gout (see online supplementary table S6), indicating population differences in the genetic basis of gout.

Taking into account the evidence of shared genetic background among gout subtypes (see online supplementary figure S3) and the presence of subtype-specific genetic factors of gout (figure 1, table 2), these results will provide helpful information for the development of novel subtype-specific genome tailor-made medicines and/or prevention for gout and hyperuricaemia.

Adaptive evolution results from adaptation to environmental changes over generations. Selection pressure analysis using SDS in the present study has elucidated which genes have been involved in adaptive evolution over the last 2000–3000 years in the Japanese population. The results revealed that the Japanese population has evolved to have higher SUA levels, which can result in gout, due to the *ABCG2* locus in addition to the already-known *ALDH2* gene. *ABCG2* is now a well-known susceptible gene for hyperuricaemia and gout, especially in the Japanese population.^31 42^ Few patients had gout before Westernisation of Japan about 150 years ago, which brought more purine-rich foods to Japan. The fact that *ABCG2* also has a relationship with SUA levels might thus have caused few problems to Japanese people until recently. The upside of SUA elevation in the Japanese population might include resistance to antioxidative stress, lower cancer risk, neuroprotective effect and longevity.^43 44^ Selection pressure analysis with other populations will also generate more information on evolutionary association with gout to elucidate this hypothesis.

In summary, we performed GWASs of all gout as well as of distinct gout subtypes, and identified multiple subtype-specific loci including four novel loci. Selection pressure analysis revealed significant enrichment of selection for the *ABCG2* and *ALDH2* loci in Japanese gout patients of each subtype. These findings will lead to elucidation of the molecular pathophysiology of each gout/hyperuricaemia subtype and the development of novel subtype-specific genome tailor-made medicine/prevention of gout and hyperuricaemia.
