# Supplementary material for: Subtype-specific gout susceptibility loci and enrichment of selection pressure on ABCG2 and ALDH2 identified by subtype genome-wide meta-analyses of clinically defined gout patients
Source: Ann Rheum Dis. 2020 Apr 1;79(5):657–65. doi: 10.1136/annrheumdis-2019-216644 (PMC7213308; doi:10.1136/annrheumdis-2019-216644)
Supplement: Supplementary data [file annrheumdis-2019-216644supp001.pdf]

Supplementary materials

Subtype-specific gout susceptibility loci and enrichment of selection pressure on *ABCG2* and *ALDH2* identified by subtype genome-wide meta-analyses of clinically-defined gout patients

A. Nakayama, M. Nakatochi, Y. Kawamura, K. Yamamoto, H. Nakaoka, *et al.*
Correspondence to H. Matsuo. (email: hmatsuo@ndmc.ac.jp).

Supplementary Figure S1 Gout classification and its pathophysiology
Supplementary Figure S2 Regional plots around *CDC42BPG* on chromosome 11q13.1 locus
Supplementary Figure S3 Genetic correlations between gout subtypes and between each subtype and serum uric acid levels
Supplementary Table S1 Clinical characteristics of participants
Supplementary Table S2 Clinical parameters of gout cases
Supplementary Table S3 Frequency of concomitant diseases in gout cases
Supplementary Table S4 Suggestive gout loci identified in the present genome-wide meta-analyses
Supplementary Table S5 Overlap between natural selection signatures and genetic risk of gout and its subtypes in the Japanese population
Supplementary Table S6 The effect of each gout locus from another trans-ancestry gout GWAS meta-analysis
Supplementary Method
References for Supplementary Method

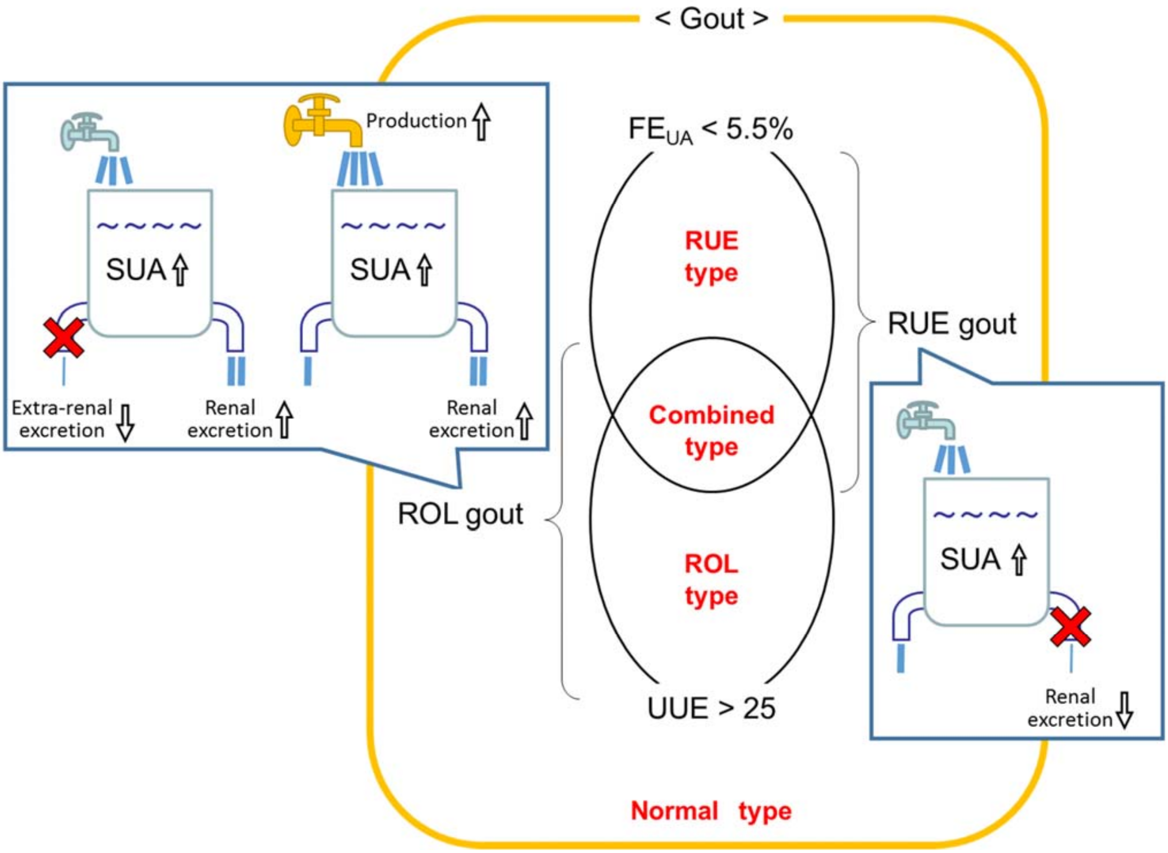

Supplementary Figure S1 Gout classification and its pathophysiology

Gout results from hyperuricemia, which is characterized by an elevated serum uric acid (SUA) level. In humans, urate is produced in organs such as the liver, and is mostly excreted from the kidney and intestine. The causes of gout can be classified into renal overload (ROL) and renal underexcretion (RUE). ROL gout (previously, “urate overproduction gout”) is caused by genuine urate overproduction and/or by extra-renal underexcretion, both of which are characterized by increased urinary urate excretion [UUE; over 25.0 mg/hr/1.73 m^2 (600 mg/day/1.73 m^2)]. On the other hand, RUE gout is caused by renal urate underexcretion, which features decreased urate clearance [urate clearance/creatinine clearance ratio, FE_{UA}; under 5.5%]. In addition to these broad subtypes (RUE gout and ROL gout), there are four distinct subtypes of gout: these consist of RUE type, ROL type, combined type and normal type gout, as shown in this Figure. These differentiated subtypes are commonly used in clinical scenarios. See also Table 1 regarding the clinical parameters of each subtype.

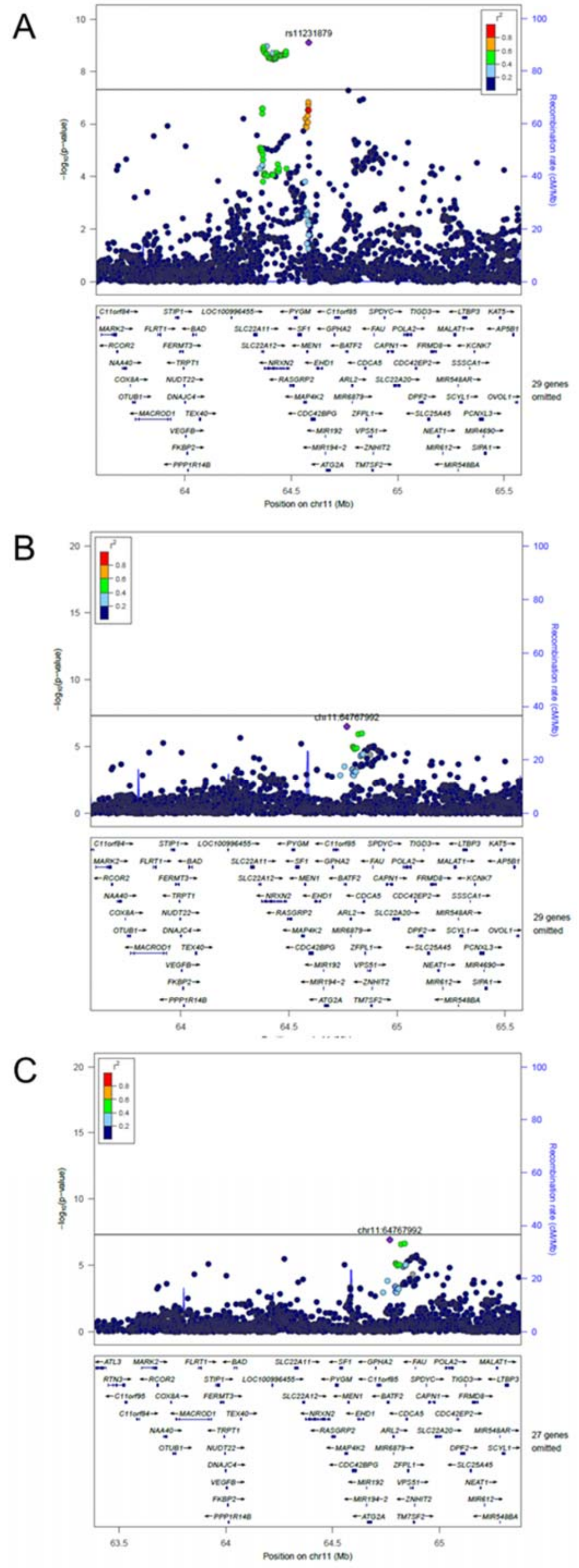

Supplementary Figure S2 Regional plots around *CDC42BPG* on chromosome 11q13.1 locus

Significance around rs11231879 of *CDC42BPG* (A) was no longer shown when conditioned on rs11231879 itself (B), nor when conditioned on the secondarily significant SNP, rs56093838 of *SLC22A12/URAT1* (C), revealing these signals to be detected from the same locus. Because URAT1 is a well-known urate transporter that markedly affects serum uric acid level, the true associated gene for combined type gout on this locus is unlikely to be *CDC42BPG*. These findings suggest that *SLC22A12/URAT1* is the actual associated gene for combined type gout on this locus (chromosome 11q13.1). Due to the lack of frequency data in 1000 genomes phase 3 JPT, r^2 data were calculated using 1000 Genomes Project Phase_3:EAS samples[1].

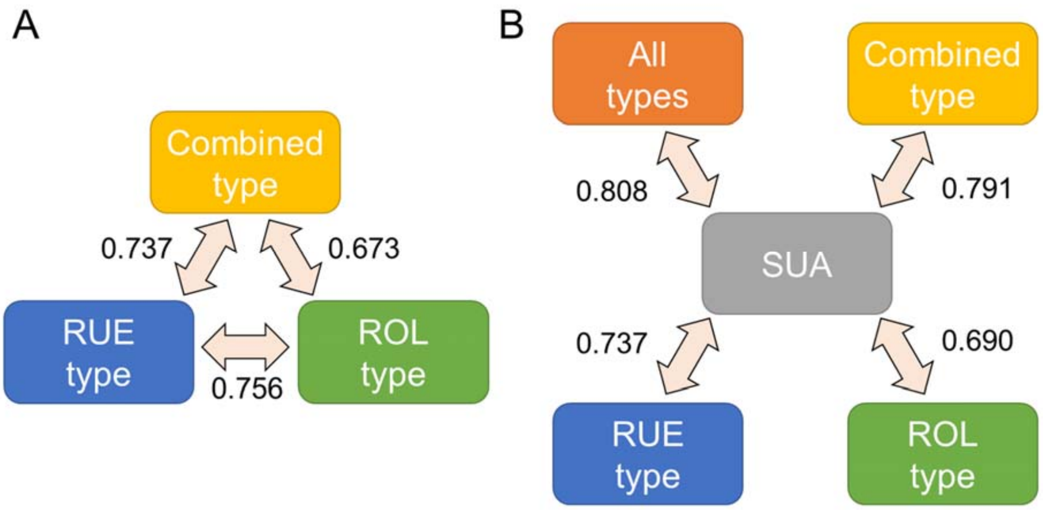

	Trait 1	Trait 2	n	r_g	SE	P
A	Combined type	ROL type	1039022	0.673	0.184	$3.00{\times}10^{-4}$
	Combined type	RUE type	1039049	0.737	0.171	$1.72{\times}10^{-5}$
	ROL type	RUE type	1039068	0.756	0.177	$2.01{\times}10^{-5}$
B	All types	SUA	1008146	0.808	0.093	$3.83{\times}10^{-18}$
	Combined type	SUA	1007682	0.791	0.123	$1.44{\times}10^{-10}$
	ROL type	SUA	1007578	0.69	0.126	$4.20{\times}10^{-8}$
	RUE type	SUA	1007663	0.737	0.114	$1.03{\times}10^{-10}$

Supplementary Figure S3 Genetic correlations between gout subtypes and between each subtype and serum uric acid levels

Strong genetic correlations were detected (A) among each subtype and (B) between subtypes and serum uric acid (SUA) level.

r_g, genetic correlation score; SE, standard error; ROL, renal overload; RUE, renal underexcretion; SUA, serum uric acid level.

Supplementary Table S1 Clinical characteristics of meta-analyzed participants

	Japonica Array platform		Illumina Array platform		Total	
	**Case**	**Control**	**Case**	**Control**	**Case**	**Control**
Number	1028	952	2025	3602	3053	4554
Age (year)	44.6 ± 11.4	54.1 ± 9.2	48.0 ± 11.8	55.5 ± 9.20	46.9 ± 11.8	55.2 ± 9.2
Body-mass index (kg/m^2)	25.2 ± 3.6	23.0 ± 2.9	25.0 ± 3.6	23.6 ± 3.0	25.1 ± 3.6	23.5 ± 3.0

Plus-minus values are means ± SD.

Supplementary Table S2 Clinical parameters of gout cases

		Number	SUA (mg/dl)	FE_{UA} (%)	UUE (mg/hr/1.73 m^2)
Japonica Array platform	RUE type	211	8.36 ± 1.28	3.78 ± 0.82	20.6 ± 3.6
	ROL type	137	8.17 ± 1.18	6.58 ± 0.96	37.6 ± 8.6
	Combined type	349	8.75 ± 1.22	4.34 ± 0.69	31.9 ± 7.3
	Normal type	16	7.45 ± 0.73	6.59 ± 1.16	20.1 ± 4.6
	Total	713	8.50 ± 1.25	4.66 ± 1.31	29.4 ± 9.3
Illumina Array platform	RUE type	443	8.38 ± 1.15	3.78 ± 0.82	20.2 ± 3.5
	ROL type	349	8.20 ± 1.14	6.89 ± 1.62	36.4 ± 9.2
	Combined type	556	8.76 ± 1.24	4.41 ± 0.72	31.6 ± 6.5
	Normal type	76	7.96 ± 1.24	6.30 ± 0.75	21.6 ± 2.8
	Total	1424	8.46 ± 1.21	4.93 ± 1.63	28.7 ± 9.2
Japonica Array platform + Illumina Array platform	RUE type	654	8.37 ± 1.19	3.78 ± 0.82	20.3 ± 3.5
	ROL type	486	8.19 ± 1.15	6.81 ± 1.47	36.7 ± 9.0
	Combined type	905	8.75 ± 1.22	4.39 ± 0.71	31.7 ± 6.8
	Normal type	92	7.88 ± 1.19	6.35 ± 0.84	21.3 ± 3.2
	Total	2137	8.47 ± 1.23	4.84 ± 1.53	28.9 ± 9.2

Plus-minus values are means ± SD.

SUA, serum uric acid; FE_{UA}, fractional excretion of urate clearance; UUE, urinary urate excretion; ROL, renal overload; RUE, renal underexcretion.

Supplementary Table S3 Frequency of concomitant diseases in gout cases

Concomitant diseases	Frequency (%)
Hypertension	27.8
Dyslipidemia	7.30
Diabetes mellitus	3.03
Ischemic heart disease	1.78
Stroke	0.59
Renal disease*	1.17

Gout cases with subtype data (n = 2137) were investigated.

*Renal disease is defined as high serum creatinine level (≥1.5 mg/dl).

Supplementary Table S4 Suggestive gout loci identified in the present genome-wide meta-analyses

SNP*	Locus	Chr.	Position (bp)†	Gene	Alleles		Illumina Array				Japonica Array				Meta-analysis			
					Risk	Non-risk	RAF		OR (95%CI)	P value	RAF		OR (95%CI)	P value	OR (95%CI)	P value	I^2	HetP
							Case	Control			Case	Control						
All gout patients																		
rs1797052	1q21.1	1	145727683	*PDZK1*	T	C	0.208	0.180	1.19 (1.07-1.31)	7.60×10^{-4}	0.228	0.176	1.39 (1.18-1.63)	5.68×10^{-5}	1.24 (1.14-1.35)	5.99×10^{-7}	61.9	0.105
rs10167307	2p12	2	75716307	*TACR1 - EVA1A*	G	A	0.415	0.376	1.24 (1.13-1.35)	3.85×10^{-6}	0.404	0.380	1.14 (1.00-1.30)	5.03×10^{-2}	1.20 (1.12-1.30)	8.59×10^{-7}	0	0.331
rs333042	8p21.3	8	19047771	*LOC100128993*	T	C	0.097	0.072	1.40 (1.22-1.62)	2.38×10^{-6}	0.096	0.079	1.24 (0.98-1.57)	7.85×10^{-2}	1.36 (1.20-1.53)	7.16×10^{-7}	0	0.376
rs147430423	10q21.2	10	63849082	*ARID5B*	G	T	0.035	0.019	2.23 (1.69-2.93)	1.34×10^{-8}	0.027	0.024	1.21 (0.79-1.85)	3.87×10^{-1}	1.86 (1.48-2.35)	1.57×10^{-7}	81.9	0.019
rs7116562	11p15.5	11	1371230	*TOLLIP - AS1- BRSK2*	C	T	0.559	0.520	1.16 (1.07-1.25)	2.37×10^{-4}	0.564	0.509	1.27 (1.12-1.44)	2.50×10^{-1}	1.19 (1.11-1.27)	4.40×10^{-7}	29.2	0.235
rs557868370	12q13.11	12	46600163	*SLC38A1*	T	G	0.043	0.032	1.77 (1.41-2.23)	8.80×10^{-7}	0.036	0.030	1.29 (0.88-1.90)	1.96×10^{-1}	1.63 (1.34-1.99)	9.97×10^{-7}	47.7	0.167
rs36045144	12q24.31	12	122618131	*MLXIP*	G	A	0.298	0.255	1.25 (1.15-1.37)	6.61×10^{-7}	0.294	0.269	1.15 (1.00-1.32)	5.58×10^{-2}	1.22 (1.13-1.32)	1.70×10^{-7}	3.5	0.309
RUE type gout patients																		
rs12035406	1q44	1	245927242	*SMYD3*	G	A	0.732	0.676	1.41 (1.18-1.67)	9.94×10^{-5}	0.725	0.648	1.46 (1.15-1.85)	1.77×10^{-3}	1.42 (1.24-1.64)	6.19×10^{-7}	0	0.801
rs1260333	2p23.3	2	27748624	*GCKR - C2orf16*	A	G	0.638	0.543	1.42 (1.22-1.64)	3.95×10^{-6}	0.630	0.570	1.28 (1.02-1.60)	2.99×10^{-2}	1.37 (1.21-1.55)	4.57×10^{-7}	0	0.452
rs142840709	3p21.31	3	47764055	*SMARCC1*	C	G	0.039	0.018	3.43 (2.13-5.53)	4.25×10^{-7}	0.038	0.023	1.82 (0.98-3.41)	5.88×10^{-2}	2.72 (1.86-3.97)	2.42×10^{-7}	59.7	0.115
rs142605903	3p14.1	3	69632183	*FRMD4B - MITF*	A	T	0.046	0.017	2.98 (2.04-4.34)	1.43×10^{-8}	0.010	0.011	0.82 (0.27-2.49)	7.29×10^{-1}	2.6 (1.82-3.72)	1.47×10^{-7}	78.5	0.031
rs6949071	7p21.3	7	13655049	*ARL4A - ETV1*	C	G	0.280	0.226	1.45 (1.21-1.74)	5.04×10^{-5}	0.287	0.222	1.55 (1.18-2.03)	1.62×10^{-3}	1.48 (1.27-1.72)	3.03×10^{-7}	0	0.702
rs149662848	7q31.1	7	109563564	*C7orf66 - EIF3IP1*	C	T	0.033	0.017	2.08 (1.36-3.19)	7.60×10^{-4}	0.049	0.018	3.59 (1.94-6.63)	4.50×10^{-5}	2.49 (1.75-3.53)	3.51×10^{-7}	50.8	0.154
rs35679068	7q36.1	7	150881888	*ASB10*	T	A	0.158	0.110	1.54 (1.26-1.88)	2.17×10^{-5}	0.146	0.105	1.49 (1.09-2.02)	1.18×10^{-2}	1.52 (1.29-1.80)	8.08×10^{-7}	0	0.857
rs6987025	8q11.23	8	52663478	*PXDNL*	T	A	0.052	0.021	2.56 (1.81-3.63)	1.28×10^{-7}	0.028	0.023	1.25 (0.64-2.43)	5.07×10^{-1}	2.19 (1.61-2.98)	6.20×10^{-7}	71.4	0.062
rs77984796	11q13.4	11	74272174	*LOC100287896 - POLD3*	C	T	0.040	0.017	2.78 (1.84-4.19)	1.18×10^{-6}	0.027	0.015	1.95 (0.91-4.16)	8.40×10^{-2}	2.56 (1.78-3.68)	3.51×10^{-7}	0	0.421

ROL type gout patients																		
rs11974814	7q22.1	7	99719504	*CNPY4*	C	T	0.191	0.131	1.74 (1.41-2.16)	2.94×10^{-7}	0.147	0.128	1.22 (0.82-1.81)	3.25×10^{-1}	1.61 (1.33-1.94)	6.22×10^{-7}	58.6	0.120
rs11201829	10q23.1	10	87671031	*GRID1*	G	T	0.037	0.015	3.86 (2.33-6.38)	1.51×10^{-7}	0.000	0.012	-	9.83×10^{-1}	3.86 (2.33-6.38)	1.51×10^{-7}	0	0.983
rs62070600	17q24.3	17	68714214	*KCNJ2 - CASC17*	G	A	0.103	0.065	1.61 (1.24-2.1)	3.62×10^{-4}	0.124	0.068	2.2 (1.43-3.38)	3.12×10^{-4}	1.76 (1.4-2.2)	8.41×10^{-7}	31	0.229
Combined type gout patients																		
rs145480657	2q21.2	2	134359704	*NCKAP5 - MIR3679*	A	G	0.039	0.015	2.83 (1.91-4.18)	1.80×10^{-7}	0.021	0.015	1.44 (0.73-2.84)	2.98×10^{-1}	2.39 (1.71-3.36)	4.51×10^{-7}	65.1	0.091
rs11099097	4q21.21	4	81167309	*PRDM8 - FGF5*	C	T	0.723	0.670	1.34 (1.15-1.55)	1.08×10^{-4}	0.732	0.665	1.41 (1.16-1.72)	6.89×10^{-1}	1.36 (1.21-1.53)	2.89×10^{-7}	0	0.662
Normal type gout patients																		
rs185157360	3q26.33	3	179058549	*ZNF639 - MFN1*	G	C	0.054	0.013	9.59 (3.99-23.07)	4.47×10^{-7}	0.001	0.016	0 (0-106952995.34)	6.35×10^{-1}	9.49 (3.95-22.82)	4.97×10^{-7}	0	0.512
rs76952758	6p21.1	6	45683258	*RUNX2 - CLIC5*	T	C	0.061	0.014	7.77 (3.44-17.52)	7.82×10^{-7}	0.017	0.012	2.44 (0.08-71.50)	6.05×10^{-1}	7.29 (3.31-16.07)	8.49×10^{-7}	0	0.514
rs150881933	6p12.1	6	56581700	*DST*	T	C	0.054	0.019	6.68 (2.79-15.99)	1.98×10^{-5}	0.095	0.025	8.36 (1.86-37.57)	5.59×10^{-3}	7.07 (3.33-15.04)	3.74×10^{-7}	0	0.800
rs118003389	7q21.11	7	81361574	*HGF*	G	C	0.144	0.047	4.23 (2.54-7.04)	2.81×10^{-8}	0.045	0.059	0.68 (0.11-4.22)	6.81×10^{-1}	3.71 (2.27-6.05)	1.63×10^{-7}	72.1	0.059
rs79022888	9q34.13	9	135005875	*MED27 - NTNG2*	T	C	0.123	0.048	3.26 (1.89-5.62)	2.18×10^{-5}	0.180	0.061	4.19 (1.49-11.81)	6.62×10^{-3}	3.44 (2.12-5.57)	5.13×10^{-7}	0	0.672
rs4558213	12q24.32	12	127291825	*LINC00944 - LINC02372*	T	A	0.730	0.553	2.42 (1.66-3.51)	3.49×10^{-6}	0.719	0.558	2.06 (0.92-4.60)	7.71×10^{-2}	2.35 (1.67-3.29)	7.33×10^{-7}	0	0.726
rs139921868	15q26.1	15	91741296	*SV2B*	C	T	0.044	0.014	4.25 (1.79-10.12)	1.07×10^{-3}	0.117	0.014	21.31 (5.11-88.92)	2.69×10^{-5}	6.56 (3.13-13.78)	6.50×10^{-7}	72	0.059
rs71651656	21q21.3	21	27140536	*GABPA*	C	T	0.052	0.012	7.44 (3.25-17.00)	1.97×10^{-6}	0.073	0.014	7.46 (1.57-35.48)	1.16×10^{-2}	7.44 (3.58-15.45)	7.23×10^{-8}	0	0.997

*dbSNP rs number.

†SNP positions are based on NCBI human genome reference sequence Build hg19.

Chr., chromosome; RAF, risk allele frequency; OR, odds ratio; CI, confidence interval; RUE, renal underexcretion; ROL, renal overload.

Supplementary Table S5 Overlap between natural selection signatures and genetic risk of gout and its subtypes in the Japanese population

Label	All associated SNPs			SNPs outside of *ALDH2*			SNPs outside of *ABCG2*			SNPs outside of *ABCG2* and *ALDH2*		
	N	SDSChi_Inflat	P value	N	SDSChi_Inflat	P value	N	SDSChi_Inflat	P value	N	SDSChi_Inflat	P value
All gout	14	3.78	1.95×10^{-6}	13	2.25	5.97×10^{-3}	13	3.42	2.61×10^{-5}	12	1.73	0.0538
RUE type	12	3.57	2.35×10^{-5}	11	1.99	0.0250	11	3.13	3.13×10^{-4}	10	1.34	0.202
ROL type	5	5.86	2.03×10^{-5}	4	3.06	0.0157	4	5.20	3.49×10^{-4}	3	1.24	0.293
Combined type	8	5.10	2.30×10^{-6}	7	3.27	1.77×10^{-3}	7	4.60	3.71×10^{-5}	6	2.39	0.0260
Normal type	10	1.50	0.133	10	1.50	0.133	9	0.72	0.693	9	0.72	0.693

SDSChi_Inflat, inflation of the selection χ^2 value of singleton density score.

Supplementary Table S6 The effect of each gout locus from another trans-ancestry gout GWAS meta-analysis

SNP*	Locus	Chr.	Position†	Gene‡	Alleles		Present meta-analysis§		Meta-analysis of gout by Tin *et al*¶.			
					Risk	Non-risk	OR (95%CI)	P value	N	RAF	OR (95%CI)	P value
rs1260326	2p23.3	2	27730940	*GCKR*	T	C	1.30 (1.21-1.39)	$2.07×10^{-13}$	721559	0.390	1.20 (1.18-1.24)	$6.40×10^{-45}$
rs3775946	4p16.1	4	9995256	*SLC2A9*	G	A	1.63 (1.52-1.75)	$3.73×10^{-41}$	763753	0.760	1.52 (1.47-1.57)	$3.19×10^{-158}$
rs4148155	4q22.1	4	89054667	*ABCG2*	G	A	2.23 (2.08-2.41)	$1.81×10^{-101}$	763813	0.111	2.04 (1.97-2.12)	$2.64×10^{-299}$
rs2817188	6p22.2	6	25807603	*SLC17A1*	G	A	1.35 (1.22-1.50)	$1.06×10^{-8}$	763774	0.553	1.18 (1.15-1.21)	$1.47×10^{-35}$
rs3129500	10q23.2	10	88915107	*SHLD2/FAM35A*	G	A	1.37 (1.28-1.48)	$4.34×10^{-17}$	259987	0.869	1.04 (0.96-1.13)	0.306
rs145954970	11q13.1	11	64273830	*SLC22A11*	C	G	12.43 (7.39-20.92)	$2.25×10^{-21}$	NA	NA	NA	NA
rs671	12q24.12	12	112241766	*ALDH2*	G	A	1.93 (1.78-2.10)	$3.19×10^{-54}$	449508	0.9999	2.47 (0.29-21.22)	0.410
**rs76499759**	**13q22.1**	**13**	**73568511**	***PIBF1***	**A**	**G**	**1.27 (1.17-1.38)**	**$2.79×10^{-8}$**	660138	0.006	1.19 (0.86-1.66)	0.287
**rs9926388**	**16p12.3**	**16**	**20558441**	***ACSM2B***	**A**	**G**	**1.24 (1.15-1.33)**	**$2.30×10^{-8}$**	763875	0.796	1.02 (0.99-1.05)	0.254
rs1010269	17q23.2	17	59448945	*BCAS3*	G	A	1.24 (1.16-1.33)	$1.81×10^{-9}$	721559	0.820	1.09 (1.05-1.12)	$3.30×10^{-6}$

*dbSNP rs number.

†SNP positions are based on NCBI human genome reference sequence Build hg19.

‡Novel loci in the present meta-analysis are shown in bold.

§The results from all gout cases are shown, since the data for subtype GWAS of gout were not available in other studies.

¶Tin *et al.* performed the trans-ancestry GWAS meta-analysis of gout, including self-reported gout cases[2], while our present study analyzed only clinically-defined gout.

Chr.: chromosome; RAF: risk allele frequency; OR: odds ratio; CI: confidence interval.

Supplementary method

Study subjects and patients' involvement

We performed subtype genome-wide meta-analyses based on two case-control data sets for clinically-defined gout that included the Japonica Array[3] and Illumina Array data sets. Patients with known clinical parameters were recruited from Japanese male outpatients at the gout clinics of Jikei University Hospital (Tokyo, Japan), Midorigaoka Hospital (Osaka, Japan), Kyoto Industrial Health Association (Kyoto, Japan), Ryougoku East Gate Clinic (Tokyo, Japan), Nagase Clinic (Tokyo, Japan), Tokorozawa Central Hospital (Tokorozawa, Japan) and Wakasa Clinic (Tokorozawa, Japan). All 3104 cases (1048 cases for the Japonica Array and 2056 for the Illumina Array) were clinically diagnosed as having primary gout according to the criteria established by the American College of Rheumatology,[4] and their subtypes were also diagnosed along with their clinical parameters as described previously[5-8] (Table 1 and Online Supplementary Figure S1). As controls, 6081 individuals (1179 and 4902 controls for the Japonica Array and Illumina Array, respectively) were assigned from Japanese male participants in the Japan Multi-Institutional Collaborative Cohort Study (J-MICC Study).[9,10] This research was done without patient involvement. Patients were not invited to comment on the study design and were not consulted to develop patient-relevant outcomes or interpret the results. Patients were not invited to contribute to the writing or editing of this document for readability or accuracy.

Genotyping and imputation for the Japonica Array data set

A total of 1048 male clinically-defined gout cases and 1179 male controls from the J-MICC Study[9,10] were genotyped with the use of a Japonica SNP Array.[3] The clustering plots were first classified by the Ps classification function in the SNPolisher package (version 1.5.2; Affymetrix). Single nucleotide polymorphisms (SNPs) that were assigned 'recommended' by the Ps classification were retained. Seven samples (two cases and five controls) with a genotype call rate of < 0.98 were excluded. Three controls with inconsistent sex information between questionnaires and the estimate from genotype were excluded. The identity-by-descent method implemented in PLINK 1.9 software[11] detected 17 duplicates or closely related pairs of samples (pi-hat > 0.1875), with one sample in each pair (15 cases and 2 controls) being excluded. One control indicated a relationship to other samples (pi-hat > 0.05), and was removed. Principal component analysis (PCA)[12] with the 1000 Genomes Project reference panel (Phase 3)[1] detected four subjects (3 cases and 1 control) with probable ancestries outside the Japanese population: these four samples were also excluded. Among the SNPs that were genotyped with the array, we excluded SNPs at the pseudo-autosomal regions of chromosome X as well as a) those with a

genotype call rate of < 0.98, b) a Hardy-Weinberg equilibrium exact test P value of $< 1 \times 10^{-6}$, c) a minor allele frequency (MAF) of < 0.01, or d) a departure from the allele frequency computed from the 1000 Genomes Project Phase 3 EAS samples[1]. This quality control filtering process resulted in the selection of 1028 case subjects and 1167 control subjects as well as 603,009 SNPs. Pre-phasing and imputation were performed using SHAPEIT2[13] and Minimac3,[14] respectively. Post-imputation quality control was performed by excluding SNPs with an imputation quality score (r^2) of < 0.3, MAF < 0.01 and indels; also excluded were 215 controls who had hyperuricemia (serum uric acid level (SUA) > 7.0 mg/dl) or a past history of gout. Ultimately, 1028 case subjects and 952 control subjects as well as 7,529,176 SNPs remained for the GWAS analysis.

Genotyping and imputation for the Illumina Array data set

As case data, 2056 male clinically-defined gout cases subjects were genotyped with the use of HumanOmniExpress or HumanOmniExpressExome BeadChip Arrays (Illumina, San Diego, CA, USA). One sample with a genotype call rate of < 0.98 was excluded. No samples showed a discrepancy between genetic and reported sex. The identity-by-descent method implemented in PLINK 1.9 software[11] detected 17 duplicates or closely-related pairs of samples (pi-hat > 0.1875), with one sample of each pair being excluded. PCA[12] with the 1000 Genomes Project reference panel (Phase 3)[1] detected six subjects who were estimated to have ancestries outside the Japanese population: these were excluded. As control data, 4902 male controls with SUA data from the J-MICC study,[9,10] who had been previously genotyped at the RIKEN Center for Integrative Medicine Sciences using a HumanOmniExpressExome BeadChip Array (Illumina, San Diego, CA, USA) with sample QC of the control data,[15,16] were used. No samples were excluded for reasons of a genotype call rate of < 0.98 or a discrepancy between genetic and reported sex. The identity-by-descent method implemented in PLINK 1.9 software[11] detected one sample having a relationship with a gout case (pi-hat > 0.1875), which was excluded. PCA[12] with the 1000 Genomes Project reference panel (Phase 3)[1] detected no subjects estimated to have ancestries outside the Japanese population. After sample QC, the case and control datasets were merged. Of the SNPs that were consistently genotyped across the arrays, we excluded SNPs at the pseudo-autosomal regions of chromosome X as well as those with a genotype call rate of < 0.98, a Hardy-Weinberg equilibrium exact test P value of $< 1 \times 10^{-6}$ or a MAF of < 0.01. This quality control filtering resulted in the selection of 2032 case subjects and 4901 control subjects as well as 553,321 SNPs. Post-imputation quality control was performed by excluding SNPs with an imputation quality score (r^2) of < 0.3, MAF < 0.01 and indels. 1062 controls with hyperuricemia (SUA > 7.0 mg/dl) or with a past history of gout were excluded. 244 samples (7 cases and 237 controls) that overlapped with the Japonica Arrays samples were

removed. Ultimately, 2025 case subjects and 3602 control subjects as well as 7,356,207 SNPs remained for GWAS analysis.

References for Supplementary Method

1. Auton, A. *et al.* A global reference for human genetic variation. *Nature* **526**, 68-74 (2015).
2. Tin, A. *et al.* Target genes, variants, tissues and transcriptional pathways influencing human serum urate levels. *Nat. Genet.* **51**, 1459-1474 (2019).
3. Kawai, Y. *et al.* Japonica array: improved genotype imputation by designing a population-specific SNP array with 1070 Japanese individuals. *J. Hum. Genet.* **60**, 581-587 (2015).
4. Wallace, S. L. *et al.* Preliminary criteria for the classification of the acute arthritis of primary gout. *Arthritis Rheum.* **20**, 895-900 (1977).
5. Dalbeth, N. *et al.* Gout. *Nat. Rev. Dis. Primers* **5**, 69 (2019).
6. Ichida, K. *et al.* Decreased extra-renal urate excretion is a common cause of hyperuricemia. *Nat. Commun.* **3**, 764 (2012).
7. Matsuo, H. *et al.* ABCG2 dysfunction causes hyperuricemia due to both renal urate underexcretion and renal urate overload. *Sci. Rep.* **4**, 3755 (2014).
8. Matsuo, H. *et al.* Genome-wide association study of clinically defined gout identifies multiple risk loci and its association with clinical subtypes. *Ann. Rheum. Dis.* **75**, 652-659 (2016).
9. Hamajima, N. & J-MICC Study Group. The Japan Multi-Institutional Collaborative Cohort Study (J-MICC Study) to detect gene-environment interactions for cancer. *Asian Pac. J. Cancer Prev.* **8**, 317-323 (2007).
10. Asai, Y. *et al.* Baseline data of Shizuoka area in the Japan Multi-Institutional Collaborative Cohort Study (J-MICC Study). *Nagoya J. Med. Sci.* **71**, 137-144 (2009).
11. Chang, C. C. *et al.* Second-generation PLINK: rising to the challenge of larger and richer datasets. *GigaScience* **4**, 7 (2015).
12. Patterson, N., Price, A. L. & Reich, D. Population structure and eigenanalysis. *PLoS Genet.* **2**, e190 (2006).
13. Delaneau, O., Zagury, J. F. & Marchini, J. Improved whole-chromosome phasing for disease and population genetic studies. *Nat. Methods* **10**, 5-6 (2013).
14. Das, S. *et al.* Next-generation genotype imputation service and methods. *Nat. Genet.* **48**, 1284-1287 (2016).
15. Hishida, A. *et al.* Genome-wide association study of renal function traits: results from the Japan Multi-Institutional Collaborative Cohort Study. *Am. J. Nephrol.* **47**, 304-316 (2018).

16. Nakagawa-Senda, H. *et al.* A genome-wide association study in the Japanese population identifies the 12q24 locus for habitual coffee consumption: The J-MICC Study. *Sci. Rep.* **8**, 1493 (2018).
